# Reply

**Published:** 1861-03

**Authors:** A. Evans


### REPLY.

BY A. EVANS, D. D. S.

It is needless to say to the Board of Trustees and the Faculty of this Institution, that we highly prize the honor this evening received. Our presence here, our toil and study has all been with this object in view.

We can not but regard these diplomas as the very best credentials we can get; earned by much hard study, and given by those who are known and honored as teachers, they speak more emphatically, and carry more weight than the single expression of any individual. We *do prize* them, and hope so to use them, that the world shall honor them. We carry with us, gentlemen, the highest recommendation you can give. It shall be our aim ever to seek the honor, prosperity and success of this our Alma Mater, feeling that in this we shall be but promoting the highest good of our profession.

By the advice of kind friends, we were directed to this door by which to enter the profession of our choice. We were told that time and money thus spent in the acquisition of knowledge would be as capital well invested—sure, if carefully attended to, hereafter to yield a rich reward—a reward in the consciousness of duty done—a reward in the confidence of our patients—a reward in the patronage of the wise and good. Thus far, the first reward is ours, and believing that the advice was wise and given for our best good, we shall strive for the other rewards promised.

Gentlemen of the Faculty, I am delegated by my classmates to convey to you our thanks for the interest you have taken in our studies—for your constant kindness, and your indefatigable labors for our advancement and the progress of true dental science. Let me assure you, that words appear cold and formal on this occasion, I can not find language sufficient to express my feelings. This winter, which in prospective was so long and irksome, you have made short and most pleasant—you have led us on from day to day, making the acquisition of science a continued pleasure—more of a luxury than a toil; you have thrown around our studies a charm which has held us fast—until time has sped away, and we are at the close of the session,—in the midst of our Commencement exercises before we have had time to weary of your instruction or realize that your lectures for this season are done. We thank you for all your instruction—we thank you for much good counsel and advice—we thank you for that kindness and courtesy shown us—and permit us to say, we thank you for making us feel that we are just commencing to grasp the knowledge we need.

We *do* thank you, that you have laid open to our view such a large field from which to gather rich materials, which we hope will be useful in our future practice. Is it so, that the more we learn, the more we see to be yet learned?

We carry with us, gentlemen of the Faculty, the fondest recollections of this our Alma Mater; and while we may not look again on these walls as students, or gather around you as pupils, seeking that instruction which we feel has been most ably given, yet we shall ere long expect to hear that these halls are more and more thronged every year with those filling our places.

Shall we not yearly visit this shrine, and renew here our pledges for the advancement of dental science, and may we not, for years to come, see our same honored Professors laying up in store for their arduous labors here a rich reward of a profession's gratitude, if not a more substantial evidence of appreciated worth?

And now, gentlemen of the Faculty, in behalf of the graduating class, I bid you farewell—and yet, as this word strikes so deeply on our hearts, let us cherish the hope that soon and often we may all meet again and again—so good-bye.

Fellow-graduates, we this evening have imposed upon us and have assumed a solemn responsibility. We not only assume the duties of Dental Practitioners, and thus give battle to dental disease—the public have a right to expect of us capabilities to meet all the exigencies which each case may demand—but the honor of our profession has been put into our hands. Is it not true that each of us should feel that we are intrusted with the character of the avocation we pursue, and this would appear to be particularly the case in all which relates to medical science. It is true that every act of charlatanism committed by any member of the profession, degrades most of all the perpetrator thereof; still the profession itself is injured in the estimation of the public.

True science must ever remain worthy of all confidence,—a host of empirics may unrobe themselves and act the charlatan, but in doing this, they do not act out the professional man. They have stepped outside of the profession, and no longer deserve any of its honors.

The day has gone by when dentistry can be classed with unlearned trades. It becomes us, then, to see that her escutcheon in our hands shall never be tarnished by any act of ours, but that our every effort shall be to elevate more and more her standard.

We go to different places of labor; we carry with us the honors of this Institution. Her motto is, "Science combats disease." Let us prove the truth of this motto, and remember our Alma Mater looks to us as sons who will in all our acts strive to advance her interests and promote her honor.

Let it never be said we are undutiful or ungrateful sons. The Alumni of the Ohio College of Dental Surgery have influence, and we shall deserve not these parchments if that influence is not felt for her good.

Fellow-students—you who are looking forward to a like realization of your hopes, and expect, when another Commencement day arrives, to occupy our places—let me, in conclusion, urge you to perseverance. Time's rapid flight will soon revolve another year; let it not be a misspent one—every day should be one of acquirement—remember that a lost day is never found. Time idled away can never be gathered up and used again. You are all longing for the duties of professional life; forget not that these duties should never be assumed before proper preparation has been made. We hope ere long to meet you as co laborers in the field of dental science. We shall greet you as brethren; and although we have little the start of you in the race for professional renown, yet we shall be most happy to share with you in all that tends to elevate dental science.

